# Effects of meal variety on expected satiation: Evidence for a ‘perceived volume’ heuristic^☆^

**DOI:** 10.1016/j.appet.2015.01.010

**Published:** 2015-06-01

**Authors:** Gregory S. Keenan, Jeffrey M. Brunstrom, Danielle Ferriday

**Affiliations:** Nutrition and Behaviour Unit, School of Experimental Psychology, University of Bristol, 12a Priory Road, Bristol BS8 1TU, UK

**Keywords:** Meal variety, Sensory specific satiety, Decision heuristics, Food familiarity, Expected satiation, Perceived volume

## Abstract

Meal variety has been shown to increase energy intake in humans by an average of 29%. Historically, research exploring the mechanism underlying this effect has focused on physiological and psychological processes that terminate a meal (e.g., sensory-specific satiety). We sought to explore whether meal variety stimulates intake by influencing pre-meal planning. We know that individuals use prior experience with a food to estimate the extent to which it will deliver fullness. These ‘expected satiation’ judgments may be straightforward when only one meal component needs to be considered, but it remains unclear how prospective satiation is estimated when a meal comprises multiple items. We hypothesised that people simplify the task by using a heuristic, or ‘cognitive shortcut.’ Specifically, as within-meal variety increases, expected satiation tends to be based on the perceived volume of food(s) rather than on prior experience. In each trial, participants (*N* = 68) were shown a plate of food with six buffet food items. Across trials the number of different foods varied in the range one to six. In separate tasks, the participants provided an estimate of their combined expected satiation and volume. When meal variety was high, judgments of perceived volume and expected satiation ‘converged.’ This is consistent with a common underlying response strategy. By contrast, the low variety meals produced dissociable responses, suggesting that judgments of expected satiation were not governed solely by perceived volume. This evidence for a ‘volume heuristic’ was especially clear in people who were less familiar with the meal items. Together, these results are important because they expose a novel process by which meal variety might increase food intake in humans.

## Highlights

• Decision heuristics may be used when estimating the expected satiation of meals.
• This study identified perceived volume as one such decision heuristic.
• The likelihood of a volume heuristic being used increased with food variety.
• Food familiarity moderated the effect of food variety on heuristic use.

## Introduction

The ‘variety effect’ describes the increases in amount of food consumed when animals (Morrison, 1974; Rogers & Blundell, 1984; Rolls, Van Duijvenvoorde, & Rowe, 1983) and humans (Bellisle & Le Magnen, 1981; Rolls et al., 1981) are exposed to multiple foods with different sensory characteristics (taste, texture, odour, and appearance). The effects of food variety on energy intake are substantial and can occur both within a single meal and across meals (Remick, Polivy, & Pliner, 2009). In non-human animals, exposure to a variety of foods increases intake by roughly 25% (McCrory, Burke, & Roberts, 2012). In humans this figure is estimated at 29% (McCrory et al., 2012).

Research investigating the effects of meal variety has tended to concentrate on the physiological and psychological processes that promote meal termination (e.g., sensory-specific satiety (Brondel et al., 2009; Raynor & Epstein, 2001; Rolls et al., 1981; Rolls, Van Duijvenvoorde, & Rolls, 1984). However, recent research suggests that meal size is very often planned, and therefore determined, in advance of eating (Fay et al., 2011; Hinton et al., 2013). A very good predictor of self-selected portion size (kcal) is the extent to which a food is expected to deliver fullness (Brunstrom, 2011; Brunstrom & Rogers, 2009; Wilkinson et al., 2012). This ‘expected satiation’ differs considerably across foods (up to a six fold difference) and is not based solely on a food's physical size (its ‘perceived volume’) (Brunstrom, Collingwood, & Rogers, 2010; Brunstrom, Shakeshaft, & Scott-Samuel, 2008; Brunstrom & Shakeshaft, 2009). Instead, the satiation that a food is expected to deliver increases with familiarity and with previous experience of eating it to fullness – a finding that is referred to as ‘expected-satiation drift’ (Brunstrom, 2011; Brunstrom, Shakeshaft, & Alexander, 2010; Hardman, McCrickerd, & Brunstrom, 2011; Irvine, Brunstrom, Gee, & Rogers, 2013). Recently, effects of variety have been explored in the context of meal planning (Wilkinson, Hinton, Fay, Rogers, & Brunstrom, 2013). Across two studies, participants selected more food for a second course when it differed in its sensory characteristics from the first course. Here, we explored the prospect that meal variety influences energy intake by moderating beliefs about the satiating capacity of a meal *before* it begins.

Evaluating the expected satiation of a single food may be relatively simple. However, with greater meal variety (e.g., at a buffet or in a multi-component meal), the task of integrating separate expectations becomes increasingly complex. Researchers have long recognised that humans respond to decision complexity (or uncertainty) by using heuristics or ‘cognitive shortcuts’ (*e.g.,* Payne, 1976; Payne, Bettman, & Johnson, 1988; Russo & Dosher, 1983; Tversky & Kahneman, 1974). A heuristic represents a simplified rule or principle that provides a quick resolution that may not be optimal. For example, purchasing a car is a complex decision. Rather than focusing on all attributes (e.g., fuel efficiency, top speed, reliability), a prospective owner may simplify the task by focusing on a single feature (e.g., fuel efficiency). Heuristics of this kind have also been shown to be used in dietary decision making. For example, Piqueras-Fiszman and Spence (2012) added weights to the base of a plastic white bowl and showed that the weight of a food can affect its expected satiety. This was the case even after controlling for all other variables (volume, visual cues, etc). For other examples of dietary heuristic use, see Scheibehenne, Miesler, and Todd (2007) and Schulte-Mecklenbeck, Sohn, de Bellis, Martin, and Hertwig (2013).

It remains unclear how prospective satiation is estimated when the underlying decision is complicated by the concurrent presentation of multiple food items (e.g., multi-item meals). However, we know that when children are unfamiliar with a food, they tend to use its perceived volume in a portion-selection task that requires them to match its expected satiation to that of another food (Hardman et al., 2011). Similarly, we reasoned that adults might also default to this strategy (a volume-based heuristic) when they are presented with complex multi-item meals.

To test this hypothesis, participants provided separate estimates of expected satiation and physical size (perceived volume) for plates of buffet foods. The amount of meal variety depicted in each plate was systematically manipulated. These data enabled us to calculate the difference between responses based on expected satiation and responses based on perceived volume at different levels of meal variety. We anticipated a smaller difference between these responses with increased meal variety.

A second aim was to establish whether food familiarity moderates the use of this volume heuristic. As we have already noted, when instructed to assess the expected satiation of an unfamiliar food, children rely on perceived volume (Hardman et al., 2011). In our study, we anticipated that unfamiliarity might play a similar role and that it might work in combination with meal variety to further encourage the use of a volume heuristic.

## Methods

### Participants

An opportunity sample of 68 participants (36 women and 32 men) assisted with this study. Vegetarians and vegans were excluded together with anyone who declared a food allergy or intolerance. In addition, we excluded anyone who had suffered from an eating disorder in the last six months. Ethical approval was granted by the University of Bristol Faculty of Science Human Research Ethics Committee.

### Stimuli

Six ‘test’ foods were selected that are commonly served at a cold buffet meal in the UK. Here we refer to a cold buffet as a table of several different types of food that are self-served and often selected in a variety of small portions, and which tend to be consumed at a party or social gathering. Specifically, we selected ‘sausage rolls,’ ‘cocktail sausages,’ ‘vol-au-vents,’ ‘scotch eggs,’ ‘cheese & pineapple,’ and ‘salmon & cream cheese blinis.’ The macronutrient composition of each food is shown in Table 1. Each contained approximately 55 kcal (±4.2 kcal).

**Fig. 1:**
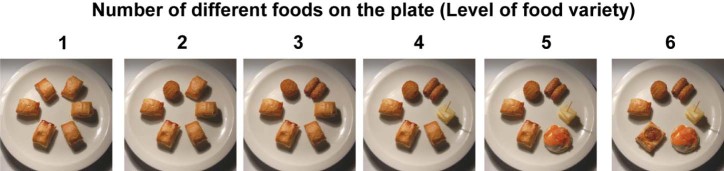
Examples of stimuli with different levels of meal variety (1–6). Level 1 has the lowest meal variety (all of the foods the same) and level 6 has the highest (six different test foods on the plate). From left to right, the stimuli match image numbers 1, 7, 13, 19, 25, and 31 in Table 2.

In each image, six test foods were arranged in isolated equally spaced positions on a 255-mm diameter white plate. Particular care was taken to ensure the lighting and viewing angle remained constant across images. Meal variety was manipulated by altering the number of different test foods on the plate. At the lowest level of meal variety, six portions of the same test food were presented. The highest level of variety comprised six different test foods. See Fig. 1 for examples. A systematic clockwise rotation method was used to ensure that each test food appeared an equal number of times across all images. This rotation method is important since it ensures that no particular test food or combination of test foods was responsible for any effects. Table 2 describes the rotation method in detail.

### Measures

#### Expected satiation

Based on previous studies (Brunstrom et al., 2010; Brunstrom & Rogers, 2009), expected satiation was assessed using a computerised ‘matched fullness’ task. In turn, each of the 36 images of buffet meals was displayed (size = 235 mm × 235 mm) on the left-hand side of a 19-inch TFT-LCD monitor. On the right-hand side, a comparison food was displayed (size = 235 mm × 235 mm). The comparison food was always rice (Ainsley Harriott, Symington's Ltd, Leeds; Nutritional information per 100 g – 111 kcal, 2.8 g protein, 23.8 g carbohydrate, 0.5 g fat, 2.3 g fibre). This food was selected because it is widely consumed in the UK. Fifty images were taken, with image 25 (250 kcal) being the midpoint and images 1 and 50 representing 50 kcal and 1250 kcal, respectively. Across this range, the portions of rice increased in equal logarithmic steps. All portion sizes were presented on the same 255-mm diameter white plate. Particular care was taken to ensure that the lighting and viewing angle remained constant across images. Participants were instructed to “look at the plate of food on the left. Now look at the food on the right. Match the picture on the right so that both foods will leave you feeling equally FULL (immediately after they have been eaten)”. Participants adjusted the amount of rice using the left and right arrow keys. Depressing the left arrow key caused a smaller portion of rice to be displayed. Depressing the right arrow key caused the converse. The pictures were loaded with sufficient speed that the change in portion size appeared to be ‘animated’. Participants were asked to press the ‘enter’ key when they had reached their desired portion size. For each pairing, this provided a ‘point of subjective equality’ (PSE), which represented the amount of the comparison (kcal) that was expected to be equally as filling as the test food. To eliminate spontaneous responding, the participants were unable to progress to the next trial until at least 30 seconds had elapsed. In each trial, the time remaining was shown at the bottom of the page and the starting rice portion was selected randomly. Across participants, the order of the trials (1–36) was also randomised.

#### Perceived volume

In all respects, this task was identical to the expected-satiation task except: i) participants were instructed to “Look at the food on the left. Now look at the food on the right. Match the picture on the right so that both foods have the same VOLUME”, and ii) the 30-second time delay was not implemented. Following Brunstrom et al. (2010), the perceived volume PSE values were also recorded in kcal to enable us to calculate the difference between matches based on expected satiation and matches based on perceived volume.

#### Food familiarity

Based on previous studies (Brunstrom et al., 2008, 2010), participants were asked to complete a food-frequency task to assess their familiarity with each of the six test foods. In turn, each meal variety level one image (six of the same test foods on a plate – see image numbers one to six in Table 2) was displayed (size = 270 mm × 210 mm), together with four different scroll boxes. Box one was headed ‘Times per day’ with response options ranging from zero to five. Box two was headed ‘Times per week’ with options zero to 35. Box three was headed ‘Times per month’ with options zero to 150, and box four was headed ‘Times per year’ with available options zero to 1000. In turn, the participants indicated how often they consumed each test food by selecting one of these response options. Selecting a response caused the totals in each box to change as appropriate. For example, if ‘one time per day’ was selected in box one, then this caused the tally in box one to change to one and also the totals associated with boxes two, three, and four to change to seven, 30, and 365, respectively. Responses were recorded as the number of times per year. Across participants, the six test foods were presented in a random order. For each participant, a composite familiarity score was calculated by summing the reported number of times per year that participants consumed each of the six test foods. We then used a median split to allocate participants to either a ‘high familiarity’ or a ‘low familiarity’ group (median = 43 occasions per year).

### Procedure

On arrival, participants read an information sheet, provided written consent and then completed the familiarity task. They then completed the expected-satiation and the perceived-volume tasks. To control for potential order effects, the presentation of these tasks was counterbalanced across participants. Before being debriefed, participants completed the restraint subscale of the Dutch Eating Behaviour Questionnaire (DEBQ) (Van Strien, Frijters, Bergers, & Defares, 1986) and a measure of age, height, and weight was obtained. Testing sessions lasted approximately 30 minutes.

### Data analysis

All data were analysed using IBM SPSS statistics, version 19 (IBM, New York, USA). In the first instance, the expected satiation (kcal) and perceived volume (kcal) scores were converted to z-scores and were screened for outliers. In a normal distribution, 99.9% of z-scores should lie between −3.29 and 3.29 (Field, 2009). Therefore, scores outside this range were entered as missing data. There were no outliers in the estimates of expected satiation. However, two participants returned perceived volume scores that exceeded 3.29.

A 2 (judgment type; expected satiation and perceived volume scores) × 6 (meal variety; levels 1–6) repeated-measures ANOVA was conducted to determine the effects of meal variety on judgments of expected satiation and perceived volume. Tukey's *post-hoc* analysis was then used to explore the interaction between judgment type and meal variety. Specifically, expected satiation and perceived volume scores were compared at each level of meal variety, separately.

To explore whether food familiarity moderated the effects of meal variety on expected satiation and perceived volume, a 2 (judgment type; expected satiation and perceived volume) × 6 (meal variety; levels 1–6) × 2 (familiarity; low or high) mixed-measures ANOVA was then used. Judgment type and meal variety were within-subjects factors and food familiarity was a between-subjects factor. To explore the three-way interaction, for the low- and high- familiarity groups separately, and for each level of meal variety, Tukey's *post-hoc* tests were used to explore differences between estimates of expected satiation and perceived volume. Note that we also ran a 2 (judgment type; expected satiation and perceived volume scores) × 6 (meal variety; levels 1–6) repeated-measures ANOVA with our continuous composite measure of familiarity included as a covariate (two familiarity scores that were greater than three standard deviations from the mean were treated as outliers and were removed from this analysis). We observed an identical pattern of data.

## Results

### Participant characteristics

Table 3 shows mean (±*S.D.*) food-familiarity scores for each test food separately. Two participants declined to report their age and one declined to have their height and weight measured. Our sample had a mean age of 31.4 years (*S.D.* = 13.1; range = 19–70) and a mean BMI of 23.0 kg/m^2^ (*S.D.* = 3.5; range = 17.2–32.6). Fourteen participants were classified as overweight and 53 as normal weight. Based on a cut-off value (median value = 2.3) on the DEBQ restraint scale (Lattimore & Caswell, 2004; Van Strien et al., 1986), our sample comprised 34 restrained and 34 unrestrained eaters.

### Effects of meal variety on judgments of expected satiation and perceived volume

**Fig. 2:**
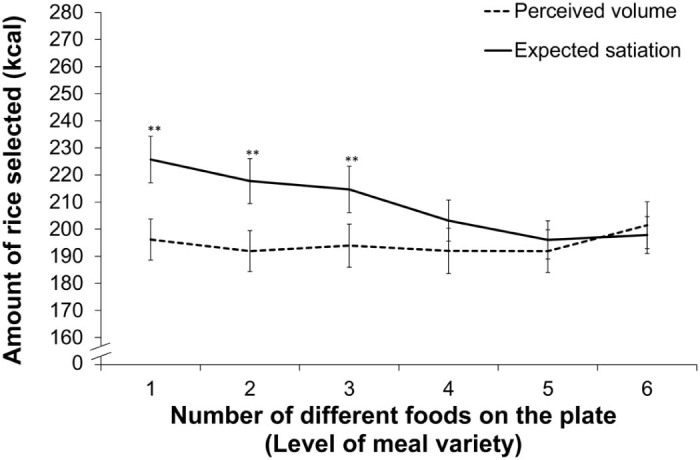
Mean (±SEM) expected satiation and perceived volume scores. Separate values are provided for scores associated with each level of meal variety (**p < .001).

Across meals, participants matched a larger quantity of rice in the expected satiation than the perceived volume task (see Fig. 2), *F(*1, 65) = 5.0, *p* = .03. We also found a main effect of meal variety – participants matched a smaller quantity of rice as meal variety increased, *F*(3.8, 246.2) = 8.5, *p* < .001.^1^ Importantly, however, we also observed a significant interaction between judgment type and level of meal variety, *F*(4.3, 281.5) = 11.5, *p* < .001.^1^ Fig. 2 illustrates this interaction. As the number of different foods on the plate increased (level of meal variety), expected satiation decreased. By contrast, perceived volume remained relatively unaffected. Indeed, Tukey's *post-hoc* tests revealed significant differences (critical difference at 5% = 13.5 and 1% = 15.4) between expected satiation and perceived volume when one, two and three different test foods were present (low variety meals). However, we found no significant differences when the level of variety exceeded three different test foods (high variety meals).

### Moderating effects of familiarity

Irrespective of the task (expected satiation or perceived volume), individuals who were more familiar with our test foods matched them to a larger quantity of rice, *F*(1, 64) = 6.2, *p* = .02.

**Fig. 3:**
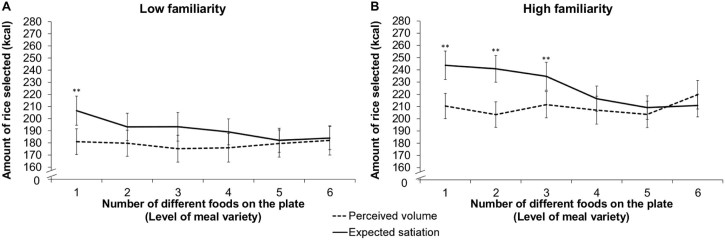
Mean (±SEM) expected satiation and perceived volume scores. Separate values are provided for scores associated with each level of meal variety (**p < .001). Respectively, low- and high- familiarity groups are represented on panels a and b.

As anticipated, we also found a significant three-way interaction *F(*4.3, 277.9) = 2.4, *p* = .04.^1^ In the high-familiarity group, we saw relatively marked differences between matches in our two tasks, especially in the low variety meals (meal variety levels one to three). By contrast, we saw much greater correspondence in the low-familiarity group (compare panels A and B in Fig. 3). This pattern is reflected in the outcome of our *post-hoc* tests. In the high-familiarity group, estimates of expected satiation and perceived volume differed significantly when one, two and three different foods were present (critical difference at 1% = 21.2, critical difference at 5% = 18.5). By contrast, in the low-familiarity group, a significant difference was only observed when a single food type was present (critical difference at 1% = 21.8, critical difference at 5% = 19.1). All other interaction terms with familiarity failed to reach significance.

## Discussion

In many cuisines, meals tend to include several items (meat, vegetables, a side dish, and so on). Despite this, very little is known about how we anticipate and plan for their combined post-ingestive consequence. In this study, we explored the hypothesis that meal variety encourages the use of a volume heuristic. We systematically manipulated meal variety and showed that it has an independent effect on judgments of expected satiation. In low variety meals, we observed a difference between estimates of expected satiation and estimates of volume. This finding is consistent with previous reports indicating that expected satiation is learned and that these judgments are not based solely on the perceived volume of a food (Brunstrom et al., 2010). By contrast, in high variety meals, we found that expected satiation was lower and that responses coincided with estimates of volume, suggesting that expected satiation was judged on this basis.

Understanding factors that govern expected satiation is important because these expectations predict self-selected portions and meal size (Brunstrom & Rogers, 2009; Fay et al., 2011; Hinton et al., 2013; Wilkinson et al., 2012). Further, food-intake studies show that variety reduces satiation (McCrory et al., 2012; Raynor & Epstein, 2001). Our work connects these observations by showing that the effect of variety is anticipated, before a meal begins. It also complements recent evidence that sensory specific satiety is anticipated and reflected in decisions about portion size, before a multi-course meal begins (Wilkinson et al., 2013). Nevertheless, future research should establish that our effects promote differences in actual food intake.

Here, we have taken a correspondence between estimates of volume and expected satiation to indicate evidence for a perceived-volume heuristic. However, we acknowledge that alternative explanations exist. In food-intake studies, meal variety is thought to increase intake because it delays the onset of sensory specific satiety (Brondel et al., 2009). One possibility is that this process is reflected in assessments of expected satiation. In other words, consciously or unconsciously, we use previous experience of the effect of variety on satiation to moderate pre-meal expectations. This process requires us to reflect on individual food items and relative differences in their sensory properties. For now, we are unable to dismiss either this ‘anticipated sensory specific satiety’ account or our ‘volume heuristic’ account with certainty. We note that these issues are further complicated by the observation that expected satiation plays a causal role in determining actual satiation at the end of a meal (Brunstrom, Brown, Hinton, Rogers, & Fay, 2011). Manipulations that increase the expected satiation of a food also enhance the satiation that it produces. If variety promotes the use of a perceived-volume heuristic (as our data suggest), and the heuristic decreases expected satiation, then this may affect actual satiation and account for the effects of variety on food intake in previous published studies.

In support of a volume heuristic, we note the following. First, we observed a correspondence between responses based on expected satiation and responses based on perceived volume. While we are careful to recognise that our findings support but do not demonstrate a causal association, it is striking how closely these two measures track one another and, in particular, in cases of high meal variety. Second, we found that familiarity moderated the expected satiation of the test foods. High familiar participants expected the test foods to deliver relatively greater satiation. This is consistent with previous robust evidence for expected-satiation drift (Brunstrom, 2011; Brunstrom et al., 2010; Hardman et al., 2011). However, we also note that familiarity moderated the level of meal variety around which we observed convergence in our measures. In highly familiar participants, we saw rather less convergence than in low familiar participants. If our effect of variety is governed by an anticipation of within-meal sensory-specific satiety, then it follows that the low familiar group expected the foods to have greater sensory diversity. We think that this is unlikely. Finally, we note that the same correspondence between expected satiation and perceived volume can be produced by exposing children to single unfamiliar foods (Hardman et al., 2011). Explanations based on variety and anticipatory sensory-specific satiety cannot account for this pattern of results. However, these observations and those in our current study can both be accommodated by a broader model of heuristic use in response to uncertainty. In our current study, we did not assess levels of certainty associated with individual judgements. Response time is commonly used as an objective measure in decision making (Festinger, 1943; Pew, 1969; Vickers & Packer, 1982). In the future, it would be interesting to incorporate this additional measure to assess confidence under different levels of variety.

In our data, variety was associated with a reduction in expected satiation. At high levels, responses in the expected satiation task coincided with those in the volume task. However, we note that our test foods were highly energy dense. Had we selected the same volume of low energy-dense foods (e.g., vegetables), then we would expect low variety meals to have lower expected satiation than their high energy-dense counterparts. By contrast, we would expect estimates of perceived volume to remain comparable, both across levels of variety and across sets of high and low energy-dense test foods. In a set of low energy-dense foods, a volume-heuristic would increase the expected satiation of high variety meals (convergence in an upward direction). This test of the volume-heuristic account is important but was beyond the scope of our study.

In the current study, food liking was not assessed. However, individuals tend to consume more of foods that they rate as highly liked (Bobroff & Kissileff, 1986; Meiselman, King, & Weber, 2003). Future studies should consider whether food liking also moderates the use of a perceived volume heuristic. In addition, there are other variables that are known to moderate the use of heuristics more generally and which might be explored systematically in this context. These include effects of time pressure (Payne et al., 1988), and the emotional valence of a decision (Tversky & Kahneman, 1981). In addition, the ‘need for closure’ (Kruglanski & Mayseless, 1987; Webster & Kruglanski, 1994), the ‘need for cognition’ (Cacioppo, Petty, & Quintanar, 1982), and the tendency to use a compensatory decision-making style (Zakay, 1990) have all been implicated as moderators of subjective task complexity, and should be considered potential candidates as additional moderators of heuristic use in complex dietary decisions.

### Conclusion

Given the popularity of multi-item meals, it is surprising that this is the first study to explore the effect of meal variety on expected satiation. This work has exposed a potential mechanism by which variety might promote increased energy intake (a volume heuristic) and it highlights several questions and areas for further research. However, it also illustrates the potential to increase expected satiation by reducing the complexity of multi-item meals.

## Figures and Tables

**Table 1:** Macronutrient composition (g) of the six test foods.

Food type	Kcal	Carbohydrate (g)	Protein (g)	Fat (g)	Fibre (g)	Weight (g)	Energy density (kcal/g)
Sausage rolls	56.0	4.7	1.3	3.6	0.4	15.5	3.6
Cocktail sausages	53.2	2.7	2.2	3.7	0.5	20.0	2.7
Vol-au-vents	57.5	6.4	0.8	3.1	0.4	20.0	2.9
Scotch eggs	55.0	3.7	1.9	3.6	0.7	20.0	2.8
Cheese & pineapple	50.8	0.7	2.9	4.0	0.0	15.5	3.3
Salmon & cream cheese blinis	53.3	5.2	5.8	1.0	0.2	38.5	1.4

**Table 2:** The rotation method that was used to allocate foods across different levels of meal variety (1–6). Each letter represents a type of food (a = sausage rolls, b = scotch eggs, c = cocktail sausages, d = cheese & pineapple, e = salmon & cream cheese blinis, f = vol-au-vents).

Image number	Degree of meal variety	Position on the plate					
		1	2	3	4	5	6
1	1	a	a	a	a	a	a
2	1	b	b	b	b	b	b
3	1	c	c	c	c	c	c
4	1	d	d	d	d	d	d
5	1	e	e	e	e	e	e
6	1	f	f	f	f	f	f
7	2	b	a	a	a	a	a
8	2	c	b	b	b	b	b
9	2	d	c	c	c	c	c
10	2	e	d	d	d	d	d
11	2	f	e	e	e	e	e
12	2	a	f	f	f	f	f
13	3	b	c	a	a	a	a
14	3	c	d	b	b	b	b
15	3	d	e	c	c	c	c
16	3	e	f	d	d	d	d
17	3	f	a	e	e	e	e
18	3	a	b	f	f	f	f
19	4	b	c	d	a	a	a
20	4	c	d	e	b	b	b
21	4	d	e	f	c	c	c
22	4	e	f	a	d	d	d
23	4	f	a	b	e	e	e
24	4	a	b	c	f	f	f
25	5	b	c	d	e	a	a
26	5	c	d	e	f	b	b
27	5	d	e	f	a	c	c
28	5	e	f	a	b	d	d
29	5	f	a	b	c	e	e
30	5	a	b	c	d	f	f
31	6	b	c	d	e	f	a
32	6	c	d	e	f	a	b
33	6	d	e	f	a	b	c
34	6	e	f	a	b	c	d
35	6	f	a	b	c	d	e
36	6	a	b	c	d	e	f

**Table 3:** Mean (±*S.D.*) food-familiarity scores (number of times consumed per year).

Food familiarity	Mean	*S.D.*
Sausage rolls	20.7	36.9
Cocktail sausages	12.9	21.4
Vol-au-vents	8.3	15.3
Scotch eggs	12.7	47.9
Cheese & pineapple	13.1	45.5
Salmon & cream cheese blinis	8.4	13.9

## Footnotes

1 A Greenhouse–Geisser correction (Greenhouse & Geisser, 1959) was applied when reporting F-values, degrees of freedom and significance since the assumption of sphericity was violated for this analysis (Mauchly's test was significant).
